# Gut Microbiota and Chronic Constipation: A Review and Update

**DOI:** 10.3389/fmed.2019.00019

**Published:** 2019-02-12

**Authors:** Toshifumi Ohkusa, Shigeo Koido, Yuriko Nishikawa, Nobuhiro Sato

**Affiliations:** ^1^Department of Microbiota Research, Juntendo University Graduate School of Medicine, Tokyo, Japan; ^2^Department of Gastroenterology and Hepatology, The Jikei University Kashiwa Hospital, Kashiwa, Japan

**Keywords:** chronic constipation, functional constipation, irritable bowel syndrome with constipation, gut microbiota, probiotics, synbiotics, antibiotics, FMT

## Abstract

**Background:** Chronic constipation, including functional constipation and constipation-type irritable bowel syndrome, is a prevalent, multifactorial gastrointestinal disorder, and its etiology and pathophysiology remain poorly understood. Recently studies using 16S rRNA-based microbiota profiling have demonstrated dysbiosis of gut microbiota in chronic constipation.

**Aims:** To provide an overview of recent studies for microbiota in chronic constipation and treatments for chronic constipation using probiotics, prebiotics, synbiotics, antibiotics and fecal microbiota transplantation (FMT).

**Methods:** PubMed searches were performed up to 1 August 2018 using keywords: “IBS,” “IBS-C,” “irritable bowel syndrome,” “irritable bowel syndrome with constipation,” “functional constipation,” “chronic constipation” in combination with “gut microbiota,” “dysbiosis,” “gut microflora” for microbiota in chronic constipation, and in combination with “probiotics,” “prebiotics,” “synbiotics,” “antibiotics,” and “fecal microbiota transplantation.”

**Results:** The findings of gut microbiota in functional constipation are inconsistent, and currently no consensus exists. Although no clear consensus exists, compared with healthy subjects, IBS-C patients have a lower level of Actinobacteria, including *Bifidobacteria*, in their fecal samples and a higher level of Bacteroidetes in their mucosa. In most randomized controlled and parallel-group trials, probiotics, prebiotics, synbiotics, antibiotics, and FMT therapy for chronic constipation were effective with few side effects. However, there are many studies in a small number and the types of probiotics are different, it is difficult to evaluate the effect.

**Conclusions:** Evidence indicates that dysbiosis of gut microbiota may contribute to functional constipation and constipation-type irritable bowel syndrome. Targeting treatments for the dysbiosis of constipation by probiotics, prebiotics, synbiotics, antibiotics, and FMT may be a new option, especially for refractory constipation to conventional therapies.

## Introduction

Recently, instead of culture methods, molecular approaches based on 16S rDNA gene sequence used to analyze gut microbiota. Advances in the culture independent technologies have shown the enormous diversity, functional capacity, and age-associated dynamics of the human microbiome. A large number of diverse microbial species reside in the distal gastrointestinal tract, and gut microbiota dysbiosis—imbalances in the composition and function of these intestinal microbes—is associated with diseases ranging from localized gastroenterological disorders including constipation to psychoneurotic, respiratory, metabolic, hepatic, and cardiovascular illnesses (1, 2).

**Figure 1:**
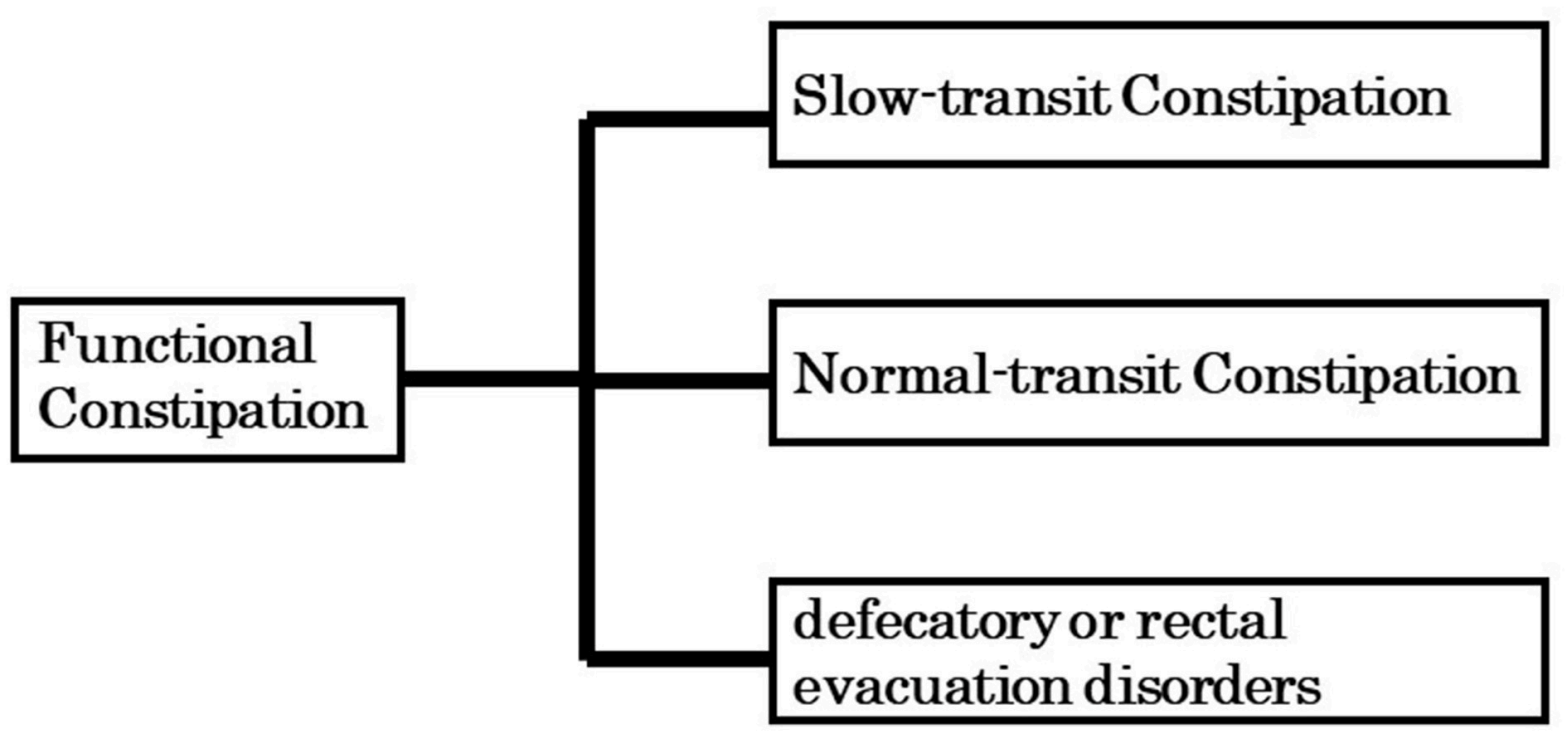
Three broad categories of functional constipation.

Functional constipation (FC: Roma classification II-IV) is typically categorized into normal transit constipation (NTC), slow transit constipation (STC), and defecatory or rectal evacuation disorders, based on specific tests such as colonic transition time, manometry evaluation and defecography (Figure 1). The defecatory or rectal evacuation disorders is caused by pelvic floor dyssynergia as well as a reduction in intra-abdominal pressure (act of bearing down), rectal sensory perception, and rectal contraction, suggesting that this type of FC is not related to gut microbiota. In contrast, NTC and STC are associated with gut microbiota. Interestingly, most literature from Western countries reports an association between STC and gut microbiota (3). Chronic constipation that is accompanied by abdominal pain is classified as irritable bowel syndrome with constipation (IBS-C: Roma classification II-IV). Gut microbiota have been shown to play a role in IBS-C.

Until recently, constipation was studied in terms of intestinal function, however many studies have revealed dysbiosis of the gut microbiota in constipated patients compared with healthy controls. In this review, we will summarize the current evidence supporting roles of the gut microbiota in the pathogenesis and treatment of chronic constipation targeting to the dysbiosis of gut microbiota.

## Gut Microbiota in Functional Constipation

Reports of dysbiosis in FC (Roma classification II–III criteria) are summarized in Table 1 (4–8). All the articles describing gut microbiota of functional constipation and chronic constipation were included (Supplemental Figure 1). Zoppi et al. (4) performed culturing analysis of fecal samples collected from children with FC (*n* = 28; mean age 9.5 y) and healthy subjects (*n* = 14; mean age 7.9 y) and demonstrated that the FC patients had a significantly higher level of *Clostridium* and *Bifidobacterium* species (*p* < 0.001 and < 0.02, respectively). They further demonstrated that *C. sporogenes, C. paraputrificum, C. fallax*, and *C. innocuum* were dominant among the *Clostridium* species. Using the same culturing methods, Khalif et al. demonstrated that patients with FC (mean age 42.2 y) had a reduced level of *Bifidobacterium, Lactobacillus, Bacteroides*, and *Clostridium* species and an increased level of Enterobacteriaceae, such as *Escherichia coli*, as well as *Staphylococcus aureus* and fungi (5). Zhu et al. (6) used 16S rRNA gene pyrosequencing to demonstrate that patients with FC (mean age 11.8 y) had a significantly lower level of Bacteroidetes, in particular *Prevotella*, and an increased level of several species of Firmicutes, including *Lactobacillus*. The authors further demonstrated that the levels of *Lactobacillus* and *Bifidobacteria* species were not reduced. Kim et al. (7) used real-time polymerase chain reaction (qPCR) to demonstrate that patients with FC (mean age 35 y) had a significantly lower level of *Bifidobacterium* and *Bacteroides* (Mann-Whitney U test; *p* = 0.030 and 0.021, respectively). Whereas, the above studies used fecal samples, more recent studies have examined mucous microbiota by biopsy samples from mucous membranes. Parthasarathy et al. (8) performed 16S rRNA metagenomics analysis (V3–V5) and demonstrated that although no difference was present in the amount of bacterial species at the genus level between FC (mean age 48 y) and healthy control groups, patients with constipation, including those with IBS, had increased levels of *Bacteroidetes* in their mucosa. However, because the constipation group in this study included 13 FC, 6 IBS-C, and 6 mixed-type IBS patients, the results are not exclusively representative of FC.

**Table 1:** Dysbiosis in functional constipation.

**References**	**Methods**	**Materials**	**Patients**	**Controls**	**Outcome**
Zoppi et al. (4)	Culture	Feces	FC children (*n* = 28) (mean age 9.5 y)	HC children (*n* = 14) (mean age 7.9 y)	FC: *Clostridium*↑ *Bifidobacterium*↑
Khalif et al. (5)	Culture	Feces	FC (*n* = 57) (mean age 42.2 y)	HC (*n* = 25)	FC: *Bifidobacterium*↓ *Lactobacillus*↓ *Clostridium* ↓ Bacteroides↓ Enterobacteriaceae (*E. coli*)↑ *S*. aureus↑ Fungi↑
Zhu et al. (6)	16S rRNA pyrosequencing	Feces	FC children (*n* = 8) (mean age 11.8 y)	HC children (*n* = 14) (mean age 13.2 y)	FC: Firmicutes↑ *Prevotella*↓
Kim et al. (7)	Quantitative RT-PCR	Feces	FC (*n* = 30) (mean age 35 y)	HC (*n* = 30) (mean age 32 y)	FC: *Bifidobacterium*↓ Bacteroides↓
Parthasarathy et al. (8)	16S rRNA Gene sequencing (V3-V5)	Feces and Mucosa	FC female (*n* = 13), IBS-C female (*n* = 6), mixed IBS female (*n* = 6) (mean age 48 y)	HC female (*n* = 25) (mean age 39 y)	FC mucosa: Bacteroidetes↑

*FC, Functional constipation; HC, Healthy control*.

These findings are inconsistent, and currently no consensus exists as to which gut microbiota are involved in FC. Because the intestinal flora is changed by age (9). However, some reports are analyzed the intestinal flora of FC in adults and the other reports are analyzed in children. Therefore, it seems to be difficult to explain the association of intestinal flora and FC.

## Gut Microbiota in Irritable Bowel Syndrome With Constipation

Reports of dysbiosis in IBS-C are summarized in Table 2 (10–15). In papers studying gut microbiota of IBS, papers reported by data on the constipation type microbiota of IBS included, and papers analyzing microbiota diarrhea type and constipation type together excluded (Supplemental Figure 2).

**Table 2:** Dysbiosis in irritable bowel syndrome with constipation.

**References**	**Methods**	**Materials**	**Patients**	**Controls**	**Outcome**
Malinenet al. (10)	Quantitative RT-PCR	Feces	IBS-C (*n* = 9) (mean age 46.5 y)	HC (*n* = 22) (mean age 45 y)	IBS-C: *Veillonella* spp↑
Maukonen et al. (11)	DGGE and Quantitative RT-PCR	Feces	IBS-C (*n* = 6) (mean age 45 y)	HC (*n* = 16) (mean age 45 y)	IBS-C: *Clostridium coccoides*-*E. rectale* group↓
Rajilić-Stojanović et al. (12)	Phylogenetic 16S rRNA microarray & Quantitative RT-PCR	Feces	IBS-C (*n* = 18) (mean age 49 y)	HC (*n* = 46) (mean age 45 y)	IBS-C: Firmicutes (*Clostridium*)↑ Bacteroidetes↓ Actinobacteria↓,
Chassard et al. (13)	Culture	Feces	IBS-C female (*n* = 14) (mean age 48 y)	HC female (*n* = 12) (mean age 30 y)	IBS-C: Enterobacteriaceae↑ Sulfate-reducing bacteria↑ Bifidobacteria↓ *Lactobacilli*↓
Durbán et al. (14)	16S rRNA gene sequencing (V1–V2)	Mucosa	IBS-C (*n* = 3) (mean age ND)	HC (*n* = 9) (mean age ND)	IBS-C: Bacteroidetes↑ Enterobacteriaceae↑
Parkes et al. (15)	FISH	Mucosa	IBS-C (*n* = 20) (mean age 32.4 y)	HC (*n* = 26) (mean age 46.1 y)	IBS-C: Bacteroidetes↑ Bifidobacteria↑ *C. coccoides*-*Eubacterium rectale*↑

*IBS-C, Constipation predominant irritable bowel syndrome; HC, Healthy control; RT-PCR, Real-time PCR; DGGE, Denaturing gradient gel electrophoresis analysis; FISH, Fluorescent in situ hybridization; ND, No description*.

Using bacterial culture tests, Malinen et al. (10) demonstrated that patients with IBS-C had significantly increased levels of *Veillonella* species compared with those of healthy controls (*p* < 0.045) as well as higher levels of *Lactobacilli* compared with those of patients with IBS with diarrhea (IBS-D) (*p* < 0.019). Maukonen et al. (11) analyzed fecal DNA using denaturing gradient gel electrophoresis (DGGE), which revealed that 30% of all bacterial species in IBS-C patients were *Clostridium coccoides* and *Eubacterium rectale*, which was significantly lower than the level in healthy control subjects (43%; p < 0.05) and IBS-D patients (50%). Using a phylogenetic 16S rRNA microarray and qPCR, Rajilić-Stojanović et al. (12) demonstrated that patients with IBS-C had a significantly higher level of Firmicutes, including *Clostridium* species (*p* < 0.05), and a significantly lower level of Actinobacteria and Bacteroidetes (*p* < 0.01) than healthy controls. In a fecal culture experiment, Chassard et al. (13) demonstrated that IBS-C patients had significantly higher levels of Enterobacteriaceae (*p* = 0.0107) and sulfate-reducing bacteria (*p* = 0.0002) and significantly lower levels of *Bifidobacteria* (*p* < 0.0001) and *Lactobacillus* (*p* = 0.0007).

In studies examining mucosal bacteria, Durbán et al. found an increased level of Bacteroidetes and Enterobacteriaceae in IBS-C patients using 16S rRNA metagenomics analysis (V1–V2) (14). In addition, Parkes et al. demonstrated that IBS-C patients had a higher level of Bacteroidetes, *Bifidobacteria*, and *C. coccoides/E. rectale* using fluorescence *in situ* hybridization (FISH) (15).

**Figure 2:**
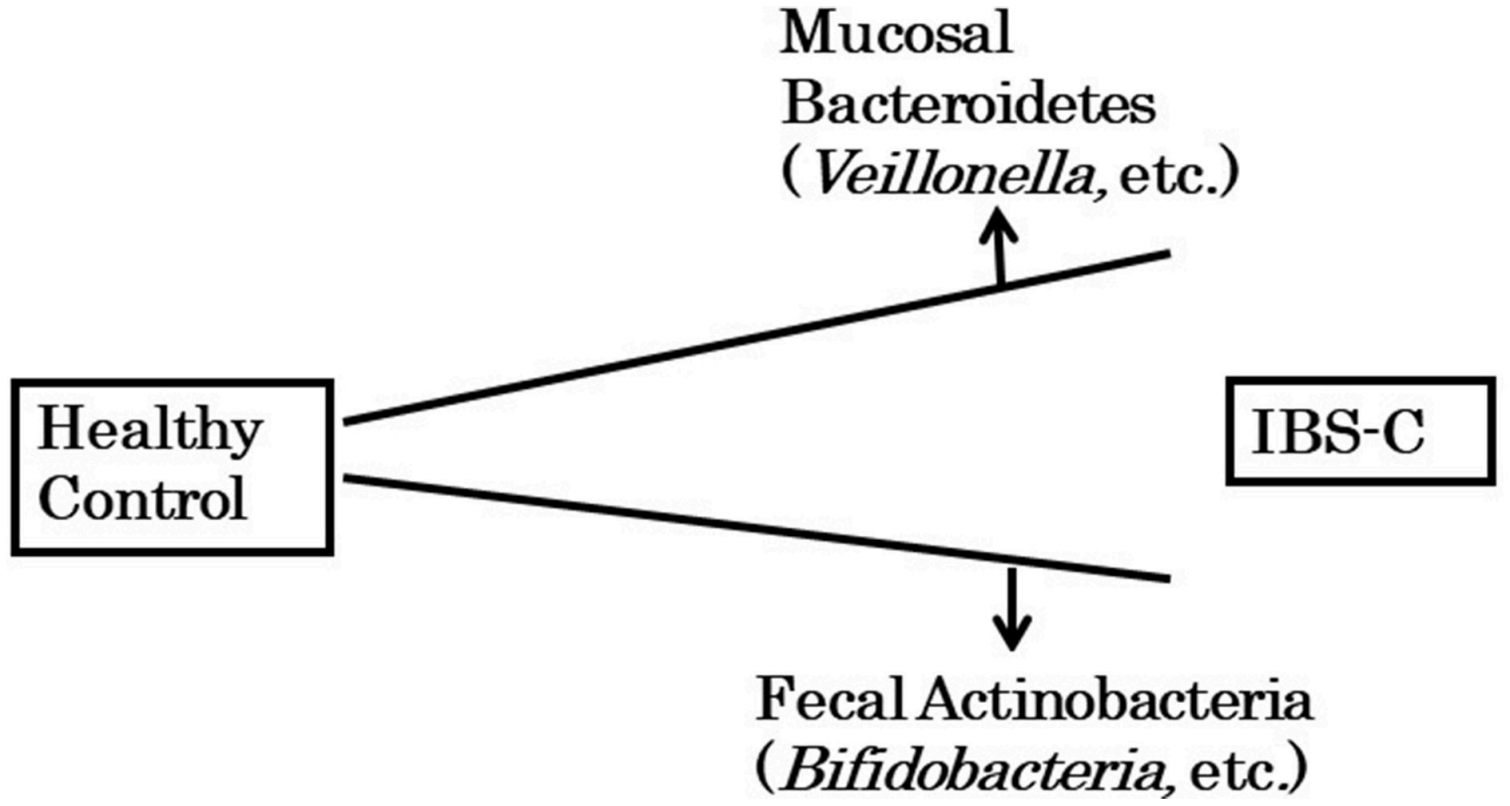
Dysbiosis in IBS-C patient.

Although no clear consensus exists, these studies suggest that compared with healthy subjects, IBS-C patients have a lower level of Actinobacteria, including *Bifidobacteria*, in their fecal samples and a higher level of Bacteroidetes in their mucosa (Figure 2). The observation that the Bacteroidetes level was high in colonic mucosa is consistent with a previous study by Parthasarathy et al. (8), which examined the mucosal microbiota in FC patients. The intestinal bacteria attached to colonic mucosa may suppress intestinal motility by metabolites produced directly or by them.

## Treatment of Chronic Constipation Using Probiotics, Prebiotics, and Synbiotics

Prebiotics are indigestible carbohydrates, such as oligosaccharide and inulin, that increase the amount of probiotics, such as *Bifidobacteria* and *Lactobacillus*, that are commonly present in humans. The algorithm the studies included in the review is shown in Supplemental Figure 3. Table 3 summarizes RCTs that have investigated the effects of these prebiotics, probiotics and synbiotics (16–37).

**Table 3:** Randomized controlled and parallel-group trials of prebiotics, probiotics and synbiotics for chronic constipation.

**References**	**Interventions (n)**	**Controls (n)**	**Prebiotics or Probiotics**	**Control materials**	**Outcome**
**PREBIOTICS**					
Bouhniket al. (16)	33 (mean age 59 y)	32 (mean age 57 y)	Lactulose	Polyethylene glycol	No significant differences
Linetzky Waitzberg et al. (17)	28 female (mean age 36.1 y)	32 female (mean age 40.2 y)	Inulin	Maltodextrin	No significant differences
**PROBIOTICS**					
Koebnick et al. (18)	35 (mean age 43.3 y)	35 (mean age 44.6 y)	*L. casei* Shirota	Beverage without probiotics	Defecation frequency↑ (*p* = 0.004) Hard stool ↓ (*p* < 0.001) Stool consistency ↓ (*p* < 0.001)
Yang et al. (19)	63 female (mean age 46.4 y)	63 female (mean age 46.4 y)	*B. lactis*	Acidified milk without probiotics	Defecation frequency↑ (*p* < 0.01) Stool consistency ↓ (*p* < 0.01) Defecation condition ↓ (*p* < 0.01)
Waller et al. (20)	High dose 33 (mean age 43 y) Low dose 33 (mean age 44 y)	34 (mean age 45 y)	*B. lactis*	Capsules with rice Maltodextrin	Abd pain↓ (*p* < 0.001) Constipation ↓ (*p* < 0.001) Irregular bowel movement ↓ (*P* < 0.01) Flatulence ↓ (p < 0.05)
Ishizuka et al. (21)	17 female (cross over) (age range 20–23 y)	17 female (age range 20–23 y)	*B. lactis*	Milk-like drink	Defecation frequency↑ (*p* < 0.05)
Riezzo et al. (22)	20 (cross over) (mean age 38.8 y)	20 (mean age 38.8 y)	*L. paracasei*	Artichokes without probiotics	Satisfactory relief of symptom (*p* = 0.0014) Stool consistency ↓ (*p* = 0.009)
Favretto et al. (23)	15 (mean age 37.5 y)	15 (mean age 40.8 y)	*B. lactis*	Fresh cheese without probiotics	Sensation of anorectal obstruction (*p* = 0.041) Defecation frequency↑ (*p* = 0.002) Constipation ↓ (*p* = 0.014)
Mazlyn et al. (24)	47 (mean age 31.8 y)	43 (mean age 31.7 y)	*L. casei* Shirota	Fermented milk without probiotics	Incomplete defecation ↓ (*p* = 0.003)
Mezzasalma et al. (25)	IBS-C, F1 50 (mean age 36.0 y) F2 50 (mean age 38.0 y)	50 (mean age 38.1 y)	F1: *L. acidophilus, L. reuteri* F2: *L. plantarum, L. rhamnosus, B. animalis* subsp. *lactis*	Inulin, silica, talc	F1, F2: Bloating↓ Abdominal pain↓ Constipation↓ Abdominal cramps↓ Flatulence↓ (*P* < 0.001)
Yoon et al. (26)	IBS-C & FC, 88 (mean age 38.3 y)	83 (mean age 39.4 y)	*S. thermophilus, L. plantarum*	Chocolate	Stool consistency ↓ (*p* = 0.002) QOL score ↑ (p = 0.044)
**PROBIOTICS IN CHILDREN**					
Bu et al. (27)	Children A: 18 (mean age 32.4 mo) Children B: 18 (mean age 36.7 mo)	Children C: 9 (mean age 35 mo)	Children A: MgO Children B: *L. casei rhamnosus*	Children C: starch	Defecation frequency↑ (*p* = 0.03) Hard stool ↓ (*p* = 0.01) Abd pain↓ (*p* = 0.03)
Coccorullo et al. (28)	Infants 22 (mean age 8.2 mo)	Infants 22 (mean age 8.8 mo)	*L. reuteri*	Identical placebo	Bowel movements↑ (*p* = 0.008~0.042)
Guerra et al. (29)	Children 30 (cross over) (age 5~15 y)	Children 29 (cross over) (age 5~15 y)	*B. longum + S. thermophilus*	*L. delbrueckii* subsp. *bulgaricus + S. thermophilus*	Defecation frequency↑ (*p* = 0.012) Defecation pain ↓ (*p* = 0.046) Abd pain↓ (*p* = 0.015)
Tabbers et al. (30)	Children 79 (mean age 7.0 y)	Children 80 (mean age 6.5 y)	*B. lactis*	Acidified milk without probiotics	No significant differences
Wojtyniak et al. (31)	Children 48 (mean age 38.7 mo)	Children 46 (mean age 37.3 mo)	*L. casei rhamnosus* Lcr35	Milk powder 99% + Mg stearate 1%	No significant differences
**SYNBIOTICS**					
Banaszkiewicz et al. (32)	Children 43 (mean age 79 mo)	Children 41 (mean age 65 mo)	*L. rhamnosus* GG + lactulose	Lactulose	No significant differences
Khodadad et al. (33)	Children B: 31 (mean age 6.2 y) Children C: 37 (mean age 5.9 y)	Children A: 29 (mean age 6.9 y)	B: *L. casei, L. rhamnosus, S. thermophilus, B. breve, L. acidophilus, B. infantis, L. bulgaricus* C: *L. casei, L. rhamnosus, S. thermophilus, B. breve, L. acidophilus, B. infantis, L. bulgaricus +* liquid paraffin	A: liquid paraffin	Defecation frequency↑ (*p* = 0.03)
Saneian et al. (34)	Children 30 (mean age 5.4 y)	Children 30 (mean age 4.7 y)	L. sporogenes + Fructooligosaccharides, Microcrystalline Cellulose, Sodium starch glycorate, Microcrystalline cellulose, sodium starch glycolate, Povidone, Hypermellose stearate, Sillicon dioxide, Propylene glycol	Paraffin	Defecation frequency↑ (*p* = 0.001) Urgency↓ (*p* = 0.010) Straining at defecation↓ (*p* = 0.004) Incomplete evacuation↓ (*p* < 0.001) Incontinence ↓ (*p* = 0.023)
Sadeghzadeh et al. (35)	Children 24 (mean age 6.1 y)	Children 24 (mean age 6.3 y)	*L. casei, L. rhamnosus, S. thermophilus, B. breve, L. acidophilus, B. infantis, L. bulgaricus +* lactulose	Lactulose	Defecation frequency↑ (*p* = 0.042) Stool consistency ↓ (*p* = 0.049)
Bazzocchi et al. (36)	17 (mean age 38 y)	12 (mean age 39 y)	Prebiotic psyllium fiber and five probiotics including *Lactobacillus* and *Bifidobacterium*	Maltodextrin	Percentage of normal stool consistency ↑ (*p* = 0.001) Intestinal transit time ↓ (*p* = 0.022)
Lim et al. (37)	43 (mean age 29.5 y)	42 (mean age 27.5 y)	Inulin-oligofructose, *L. plantarum, B. lactis*	Maltodextrin	No significant differences

Bouhnik et al. (16) administered lactulose (β-D-galactopyranosyl-(1→4)-D-fructose), an indigestible carbohydrate, and demonstrated that it was as effective as polyethylene glycol (a laxative) in relieving constipation. They further examined fecal samples of these patients and demonstrated a significant increase in the level of *Bifidobacteria* in the lactulose group (*p* = 0.04), whereas in the polyethylene glycol group showed no increase in *Bifidobacteria*, and the total amount of bacteria was decreased. In a study by Linetzky Waitzberg el al. (17), inulin was shown to have the equivalent effect of a placebo (maltodextrin) on constipation. They further demonstrated that patients who received inulin but not maltodextrin exhibited a decrease in *Clostridium* species in their fecal samples. However, because maltodextrin contains indigestible components, it may not have been an appropriate placebo.

*Lactobacillus* and *Bifidobacteria* are commonly used as probiotics in adults (18–26) and in children (27–31). Koebnick et al. (18) performed the first RCT in 2003, in which the *L. casei* Shirota strain (LcS) was administered to 35 patients with chronic constipation. Compared to placebo (*n* = 35), patients who received LcS had a significantly improved defecation frequency and stool consistency 2 weeks after administration (*p* < 0.0001). At the 5-week post-administration endpoint, the LcS group demonstrated significant improvement compared to the placebo control, with 89 and 56% improvement, respectively (*p* = 0.003). As seen in Table 3, all studies in adults indicated the effectiveness of probiotics for treating constipation. Ishizuka et al. performed a crossover study by administering either 10^10^ cfu/100 mL of *B. lactis* GCL2505 or a milk product without bacteria as a placebo in 17 patients with constipation. Two weeks after administration, patients who received *B. lactis* had a significant increase in defecation frequency and the amount of stool (19). Because the patients had a higher quantity of *B. lactis* in their feces, the authors concluded that bacterial growth may have contributed to the improvement in constipation symptoms. Recently, Yoon et al. conducted RCT by administering 3.0 × 108 CFU/g of Streptococcus thermophilus MG510 and 1.0 × 108 CFU/g of *Lactobacillus plantarum* LRCC5193 with 171 cases in many cases (26). They concluded that the probiotics significantly ameliorated stool consistency in patients with chronic constipation. In addition, the beneficial effect of *L. plantarum* on stool consistency remained after the probiotic supplementation was discontinued. In children, constipation is a clinically significant problem, and many RCTs by administration of probiotics are done. Bu et al. evaluate the efficacy of probiotics (*Lactobacillus casei rhamnosus*, Lcr35) for treating children with chronic constipation and to compare its effect with magnesium oxide (MgO) and placebo (27). They reported that Lcr35 was effective in treating children with chronic constipation. There is no statistically significant difference in efficacy between MgO and Lcr35, but less abdominal pain occurred when using Lcr35. However, recent RCT studies by Tabbers et al. (30) and Wojtyniak et al. (31) reported no significant effect of *Bifidobacterium lactis* and Lcr35 for functional constipation when compared with placebo.

Bazzocchi et al. (36) described a synbiotic treatment regimen, in which plantain fiber was used as a prebiotic in addition to a probiotic cocktail containing five species of live *Lactobacillus* and *Bifidobacteria*. They administered either the synbiotic (*n* = 17) or maltodextrin (placebo) (*n* = 12) for 8 weeks and demonstrated that patients receiving the synbiotic had a significant improvement in defecation frequency and stool consistency (*p* = 0.001). In addition, the colonic transition time was significantly reduced in the synbiotic group (*p* = 0.022), and the five species of *Lactobacillus* that were administered, including *L. planetarium, L. acidophilus*, and *L. rhamnosus*, were found in the fecal samples of half of the patients in the synbiotic group. Khodadad et al. (33) and Sadeghzadeh et al. (35) done RCT of constipation treatment of the same multiple kinds of probiotics administration (*Lactobacillus casei, Lactobacillus rhamnosus, Streptococcus thermophiles, Bifidobacterium breve, Lactobacillus acidophilus, Bifidobacterium infantis, and Lactobacillus bulgaricus*). The studies showed that synbiotics have positive effects on symptoms of childhood constipation without any side effects. However, Branaskiewicz et al. (32) and Lim et al. (37) reported that synbiotics were not an effective in treating constipation. They suggested that the result was due to the high placebo effect which synbiotics failed to demonstrate benefit over the controls.

Few side effects such as abdominal pain, abdominal distention, vomiting (16, 30, 32) were reported in the studies described above, indicating that administration of these agents is safe. Therefore, probiotics, prebiotics, and synbiotics may be effective treatment options for constipation. Because there are many studies in a small number and the types of probiotics are different, it is difficult to evaluate the effect. Therefore, it will be necessary to conduct a number of studies on specific probiotics.

## Treatment of Chronic Constipation Using Antimicrobial Agents

Previous studies have proposed a link between constipation and methanogenic bacteria via the hypothesis that methanogenic gut microbiota lead to the development of constipation by reducing bowel movement (38). Studies have shown that patients with chronic constipation have more methanogenic bacteria than healthy subjects (39, 40).

In a study by Low et al. (41), IBS-C patients with >3 ppm methane production were treated with rifaximin and neomycin for 10 days, and the changes in symptoms and breath testing outcomes were examined using lactulose breath testing. Patients received neomycin and rifaximin (*n* = 27), neomycin alone (*n* = 8), or rifaximin alone (*n* = 39). Symptoms improved in 85, 63, and 56% of patients, respectively, demonstrating a significant improvement in the neomycin + rifaximin group compared with the rifaximin alone group (*p* = 0.01). Furthermore, patients who received neomycin + rifaximin had a significantly reduced level of methanogenic bacteria. Eighty-seven percent of patients had a methane production level of < 3 ppm, as measured by breath testing, compared to 33% in the neomycin group (*p* = 0.001) and 28% in the rifaximin group (*p* = 0.001). Therefore, the authors concluded that the combination of rifaximin and neomycin was effective in the treatment of IBS-C associated with methane production (37). This study presented interesting findings that support the association between constipation and methanogenic bacteria.

## Treatment of Chronic Constipation by Fecal Microbiota Transplantation

The algorithm the studies included in the review is shown in Supplemental Figure 4. Table 4 summarizes studies (42–47) on the treatment of refractory constipation by fecal microbiota transplantation (FMT). Borody et al. (42) performed FMT on 4 patients with chronic constipation and demonstrated immediate improvements in symptoms, such as abdominal pain, early satiety, and nausea, as well as a significant improvement in defecation frequency to once or twice daily. Furthermore, in a case report of a patient with refractory constipation, the authors demonstrated that FMT effectively induced defecation 2–3 days after the transplant, with a defecation frequency of once or twice daily (43). Ge et al. (44) performed FMT on 6 patients with STC and demonstrated a significant increase in defecation frequency, from 1.6 ± 0.2 times a week at pretreatment to 5.0 ± 0.4 times a week at 12 weeks post-treatment (*p* < 0.001), as well as in stool consistency from 2.0 ± 0.3 at pretreatment to 3.3 ± 0.2 at 12 weeks post-treatment (*p* = 0.0025). They also performed 16S rRNA metagenomics analysis on fecal microbiota prior to FMT and demonstrated that patients with constipation had an increased quantity and diversity of bacteria compared to healthy subjects. However, they did not perform this analysis following FMT to detect changes that may have occurred in the fecal microbiota. Tian et al. (45) performed an RCT in which 60 patients with STC were divided into either the FMT or control group and received a conventional laxative. The comparison of the FMT (*n* = 25) and control (*n* = 24) patients who completed the study demonstrated that those who underwent FMT had significantly improved symptoms, including defecation frequency and fecal properties. Therefore, they concluded that FMT for constipation was effective, although the sample size was small. However, it is unlikely that FMT will be considered as a first choice given the challenges in identifying donors as well as the cost and complexity of the procedure. FMT should be selectively performed in patients who are refractory to conventional therapeutic strategies.

**Table 4:** Fecal microbiota transplantation (FMT) for chronic constipation.

**References**	**Study**	**Interventions (n)**	**Controls (n)**	**Follow-up Period**	**Outcome**
Borody et al. (42)	Case series	4	–	28 months	Defecation frequency↑
Andrews et al. (43)	Case report	1	–	18 months	Defecation frequency↑ Melanosis coli disappeared
Ge et al. (44)	Case series	6	–	12 weeks	Defecation frequency↑ (*P* < 0.05) Stool consistency ↓ (*P* < 0.05) Colonic transit time ↓ (*P* < 0.05)
Tian et al. (45)	RCT	30	30	12 weeks	Defecation frequency↑ (*p* = 0.001) Stool consistency ↓ (*P* < 0.00001) Colonic transit time ↓ (*P* < 0.00001) Clinical cure rate ↑ (*p* = 0.04) Clinical improvement rate ↑ (*p* = 0.009)
Ding et al. (46)	Case series	52	–	24 weeks	Defecation frequency↑ (*P* < 0.01) Stool consistency ↓ (*P* < 0.01) Incompleteness of evacuation ↓ (*P* < 0.01) Colonic transit time ↓ (*P* < 0.01)
Zhang et al. (47)	Case series	29	–	1 year	Complete spontaneous bowel movement, stool consistency, the Wexner constipation scale and constipation symptoms improved

*RCT, Randomized controlled trials*.

## Conclusions and Future Perspectives

Constipation is a syndrome indicating various and complex combinations of disorders. Given its complexity, clinical trials, such as those performed for single diseases, may not be possible. Most studies use the Rome criteria to diagnose constipation and perform functional tests to selectively target patients with STC for trials. Results from clinical studies on probiotics and FMT suggest that constipation is caused by dysbiosis of the microbiota. Thus, future studies should be performed by first categorizing constipation to identify the target population. In addition, studies on gut microbiota may identify bacterial species that promote the development of constipation. Ultimately, the identification of causative bacteria of constipation may lead to the development of probiotic, prebiotic, and synbiotic treatments that can cure constipation in the future.

## Author Contributions

TO, SK, and NS wrote and edited the manuscript. TO created the figure and critically revised the manuscript. YN gathered the documents and created tables. All authors read and approved the final manuscript for publication.

### Conflict of Interest Statement

The authors declare that the research was conducted in the absence of any commercial or financial relationships that could be construed as a potential conflict of interest.
