# Supplementary figures and images for: Gut Microbiota and Chronic Constipation: A Review and Update
Source: Front Med (Lausanne). 2019 Feb 12;6:19. doi: 10.3389/fmed.2019.00019 (PMC6379309; doi:10.3389/fmed.2019.00019)

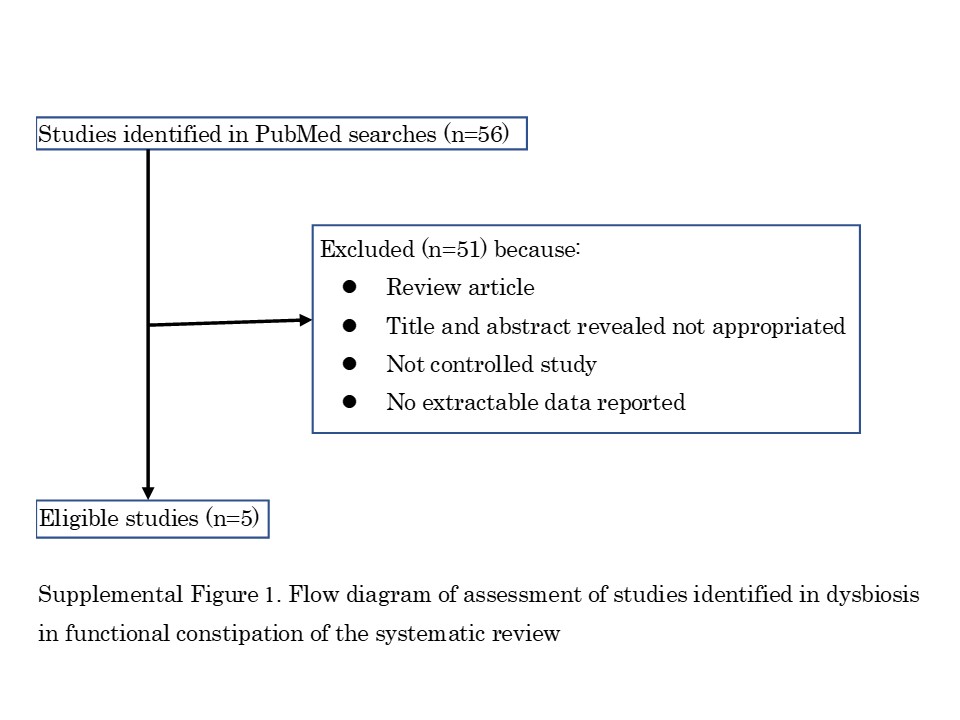

Supplement: Supplementary file 1 [file Image_1.jpg]

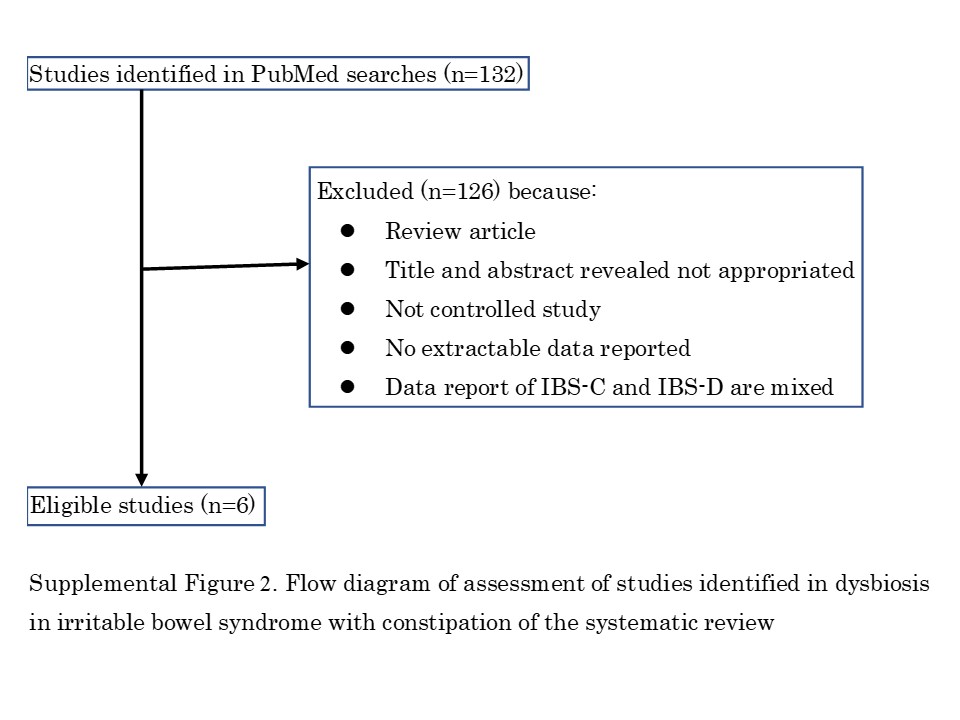

Supplement: Supplementary file 2 [file Image_2.jpg]

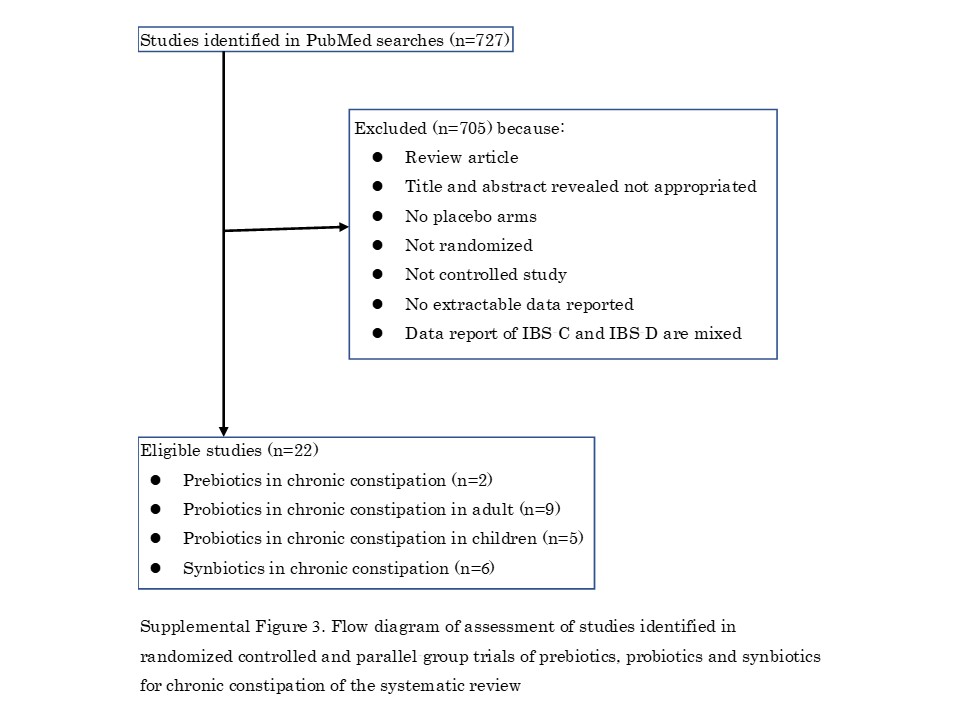

Supplement: Supplementary file 3 [file Image_3.jpg]

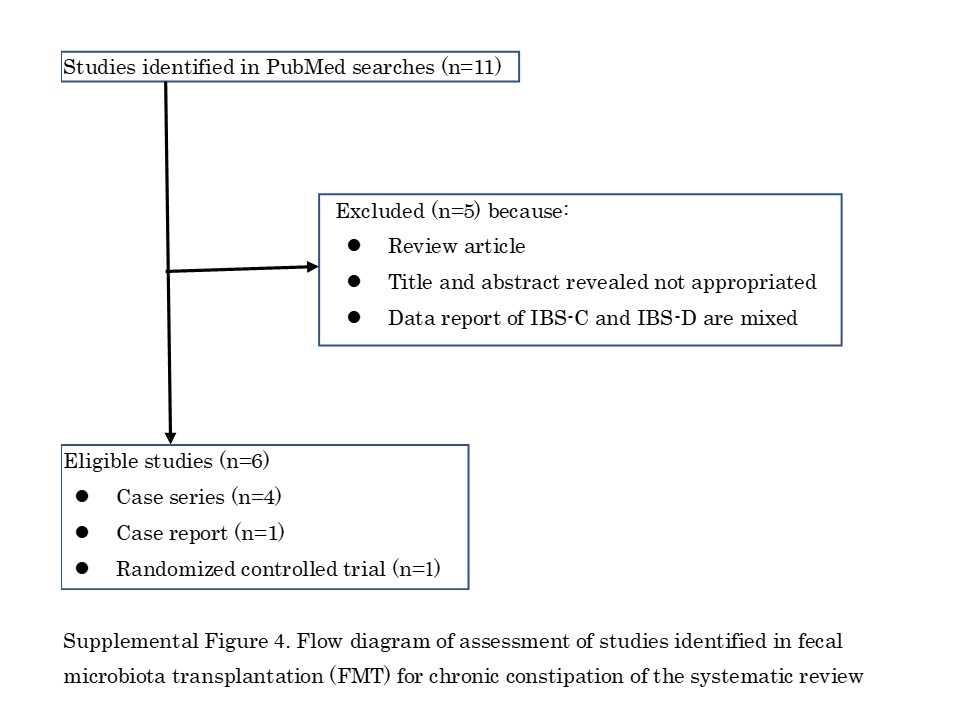

Supplement: Supplementary file 4 [file Image_4.jpg]
